# Do nutritional assessment tools (PNI, CONUT, GNRI) predict adverse events after spinal surgeries? A systematic review and meta-analysis

**DOI:** 10.1186/s13018-024-04771-3

**Published:** 2024-05-12

**Authors:** Zhi Huang, Hanbo Wang, Yifeng Da, Shengxiang Liu, Wenkai Zheng, Feng Li

**Affiliations:** The Second Affiliated Hospital of Inner Mongolia Medical University, Hohhot, Inner Mongolia China

**Keywords:** Spinal surgery, Nutritional index, Controlling nutritional status, Geriatric nutritional risk index, meta-analysis

## Abstract

**Background:**

Nutritional assessment tools are used to predict outcomes in cancer. However, their utility in patients undergoing spinal surgery is unclear. This review examined if prognostic nutritional index (PNI), controlling nutritional status (CONUT), and geriatric nutritional risk index (GNRI) can predict adverse events after spinal surgeries.

**Methods:**

PubMed, CENTRAL, Scopus, and Embase were screened by two reviewers for relevant studies up to 26th January 2024. The primary outcome of interest was total adverse events after spinal surgery. Secondary outcomes were surgical site infections (SSI) and mortality.

**Results:**

14 studies were included. Meta-analysis showed that while reduced PNI was not associated with an increased risk of SSI there was a significant association between PNI and higher risk of adverse events. Meta-analysis showed that high CONUT was not associated with an increased risk of complications after spinal surgeries. Pooled analysis showed that low GNRI was associated with an increased risk of both SSI and adverse events. Data on mortality was scarce.

**Conclusions:**

The PNI and GNRI can predict adverse outcomes after spinal surgeries. Limited data shows that high CONUT is also associated with a non-significant increased risk of adverse outcomes. High GNRI was predictive of an increased risk of SSI. Data on mortality is too scarce for strong conclusions.

**Supplementary Information:**

The online version contains supplementary material available at 10.1186/s13018-024-04771-3.

## Introduction

Spinal disorders like degenerative disease, deformities, tuberculosis, and cancer metastasis often require surgical intervention for appropriate management. Trends indicate that with increasing life expectancy there has been an upward trend in the number of spinal diseases and a significantly high number of patients require surgical procedures [1]. Advances in medical technology have led to the development of minimally invasive spinal surgeries and intraoperative navigation systems which aim to reduce surgical trauma and postoperative adverse events [2, 3]. Nevertheless, open procedures are still the cornerstone of spinal surgeries and are associated with a high risk of complications. On average, orthopaedic surgeries have a complication rate of 5%, while in spinal surgeries the figure ranges from 7–20% [4, 5]. Given the high rates, there has been an unrelenting effort to reduce complications after spinal surgeries and identify risk factors that can be modified preoperatively to improve patient outcomes.

In the past decade, nutrition has been recognized as an important factor influencing the outcomes of various diseases [6–12]. Malnutrition has been associated with overall survival and recurrence-free survival after several malignancies [7, 8, 10]. Similarly, preoperative malnutrition has been a risk factor for poor outcomes after hip surgeries, cardiovascular surgeries, and percutaneous coronary interventions [13–15]. Nevertheless, quantifying and screening for malnutrition in a surgical patient has been a challenge. Currently, the nutritional index (PNI), controlling nutritional status (CONUT), and geriatric nutritional risk index (GNRI) are some of the most commonly used nutritional assessment tools for the prognostication of patients [12]. The PNI measures the nutritional and immune status of the patient by summing the serum albumin and total lymphocyte counts [16]. CONUT improves over PNI by including cholesterol levels while GNRI is calculated by combining albumin and adjusted body weight [12]. All three indices have been well established in predicting the prognosis of various diseases [6–12], however, their utility in the assessment of adverse events after spinal surgeries is relatively unclear. In this review, we examined the utility of PNI, CONUT, and GNRI in predicting adverse outcomes after spinal surgeries.

## Materials and methods

### Criteria for selection

All observational studies published as abstract or full-text and examining the association between PNI, CONUT, GNRI, and adverse events after any type of spine surgery were eligible. In PECOS format, studies were to be on adult spinal surgery patients (*Population*) with an *Exposure* group of low PNI, high CONUT, or low GNRI *Compared* with high PNI, low CONUT, or high GNRI respectively. *Outcomes* were any peri- or postoperative adverse events. We did not define the cut-off from high and low values and all values used by the studies were acceptable. Studies reporting data on per unit increase or decrease were also included. No limitation was placed on the type of adverse events. Non-English language studies, unpublished data, and studies not reporting the ratio of the outcome or not providing data to calculate the odds ratio (OR) were excluded.

The primary outcome of interest was total adverse events after spinal surgery. The secondary outcomes were surgical site infections (SSI) and mortality.

### Identification of studies

We initially registered the review protocol on PROSPERO (Registration number CRD42024505323). Four online databases PubMed, CENTRAL, Scopus, and Embase were screened by two reviewers independently. The keywords used were: “spine surgery”, “spinal surgery”, “lumbar fusion”, “spinal tuberculosis”, “spinal deformity”, “cervical decompression”, “lumbar decompression”, “prognostic nutritional index”, “controlling nutritional status”, and “geriatric nutritional risk index”. Search queries were generated common to all databases (Supplementary material 1). No filter was applied for language, publication time, and study design. Studies published up to 26th January 2024 were included. Google Scholar was explored as a source of gray literature for any missed studies. Authors of abstracts were to be contacted for complete information. Bibliographic data of recent review articles was also scanned for any pertinent studies.

### Selection of studies

The authors complied with the PRISMA statement reporting guidelines [17]. All searched articles were imported and deduplicated using EndNote software. Both authors conducted inclusive screening to exclude non-relevant articles. The remaining studies were further reviewed by reading their titles and abstracts and refined to include only those resembling the inclusion criteria. Complete texts of the selected articles were screened further. The two reviewers examined each article based on eligibility criteria. Articles with completely overlapping articles were excluded. In case of incomplete information, the corresponding author was contacted once via email. Discrepancies were resolved by consensus. The references list of eligible articles was hand-searched for additional articles.

### Data management and study quality

information on the author’s last name, year of publication, study location, study type, surgery type, sample size, age, gender, nutritional tool used, its cut-off, method to determine cut-off, percentage with malnutrition, adjusted factors, outcomes reported, follow-up and outcome data were extracted. Data was obtained from the studies by two reviewers independently.

Data quality was appraised independently by two reviewers using the Newcastle Ottawa Scale (NOS) [18]. The NOS examines each study for selection of cohort, their comparability, and assessment of outcomes.

### Statistical analysis

Data analysis was done on “Review Manager” (RevMan, version 5.3; Nordic Cochrane Centre (Cochrane Collaboration), Copenhagen, Denmark; 2014). We extracted all outcome data from the studies in a tabular form. Data was then segregated based on the type of nutritional assessment scale. If adjusted ORs were available then they were extracted as it is. In case of missing data, ORs were calculated using standard methods. We performed a meta-analysis if there were ≥ 3 studies on the same outcome, else a descriptive analysis was done. The ORs and 95% confidence intervals (CI) were then pooled using the generic inverse variance function of RevMan. Methodological heterogeneity between studies prompted us to use the random-effect meta-analysis model. Different analysis was conducted for ORs calculated based on per unit value and those based on pre-determined cut-offs. Funnel plots were not drawn due to a limited number of studies. The I^2^ statistic was the tool to determine inter-study heterogeneity. I^2^ < 50% meant low and > 50% meant substantial heterogeneity. Sensitivity analysis was done for meta-analyses with > 3 studies.

## Results

**Fig. 1:**
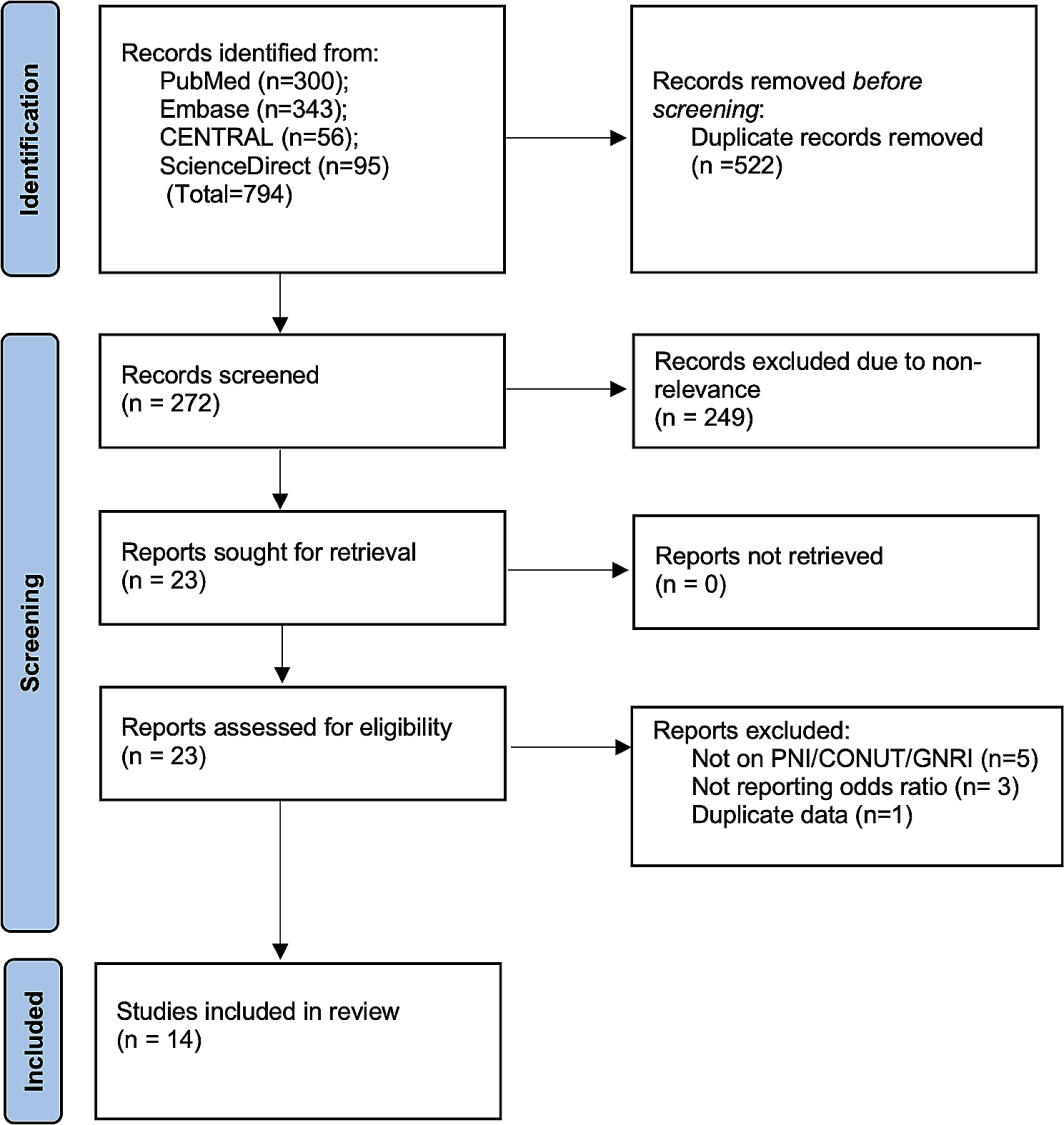
Study flow chart

Following the literature search, 794 studies were obtained (Fig. 1). Duplicates were excluded leaving 272 results. Further, 249 articles were removed due to non-relevance. 23 studies underwent full-text analysis and 14 were selected [19–32].

All fourteen studies were retrospective observational studies carried out in China, Japan, the USA, and Turkey. All were published recently between 2020 and 24. Four studies included patients undergoing all types of major spinal surgeries while the remaining included patients undergoing surgery for spinal tuberculosis, metastatic disease, and deformity. Five studies were only on PNI, four only on GNRI, and one only on CONUT. The remaining studies examined more than one nutritional tool. Most studies reported adjusted data on the association between nutritional index and adverse events while four reported unadjusted data. The follow-up of the studies varied from one month to one year. The NOS score awarded was either 7 or 8 (Table 1).

**Table 1:** Information obtained from included studies

Study	Location	Surgery	Sample size	Male gender	Mean age (years)	Tool used	Cut-off	Percentage with malnutrition	Method to determine cut-off	Adjusted factors	Outcomes	Follow-up	NOS score
Wang 2024 [19]	China	Thoracolumbar fusion	228	NR	NR	PNI GNRI	50 98	28 42.4	Standard value	Male, coronary heart disease, gastrointestinal disorders, old cerebral infarction, operative time, blood loss	Complications	3 months	8
Yang 2023 [28]	China	Posterior lumbar interbody fusion	766	401	57	PNI	NR	NR	NR	Age, gender, diabetes mellitus, ASA, length of procedure, length of fusion, hypertension, and relative fat thickness	SSI	NR	7
Rigney 2023 [23]	USA	For metastatic spine tumors	154	94	63.2	PNI	40.4	NR	Maximally selected log-rank statistics	Age, sex, primary tumor pathology, physical function, nutritional status, and frailty	Complications	6 months	8
Ramos 2023 [32]	USA	For metastatic spine tumors	139	82	63	PNI CONUT	NR	NR	NR	Age, ECOG performance status, non-ambulatory status at presentation, lung cancer, prostate cancer, and pathological vertebral compression fracture;	Mortality, SSI	3 months	8
Huang 2023 [20]	China	Spinal tuberculosis surgery	91	44	70.5	GNRI	98.63	34.4	ROC	NR	Complications, SSI	1 year	8
Mendiratta 2023 [30]	USA	Posterior cervical decompression with or without fusion	7597	4405	73	GNRI	98	15.6	Standard value	Age, body mass index, inpatient status, hemiplegia, > 10% weight loss in 6 months, cancer, pulmonary, cardiac, renal comorbidities	SSI, Mortality	1 month	7
Miura 2022 [21]	Japan	Cervical spine surgery	261	172	63	CONUT	2	36	ROC	Diabetes, ASA physical status, operative time, surgical Apgar score, combined anterior–posterior surgery and multisegment surgery	Complications	NR	7
Cao 2022 [26]	China	Spinal tuberculosis surgery	97	52	53.9	PNI CONUT	38.6 5	26.8 37.1	ROC	NR	Complications, SSI	NR	7
Ushirozako 2021 [22]	Japan	All spine surgeries	1115	369	32	PNI	NR	NR	NR	Age, sex, and diabetes mellitus	SSI	1 year	8
Li 2021 [25]	China	Posterior lumbar arthrodesis	252	110	76.8	GNRI	98	61.5	Standard value	NR	SSI	NR	7
Kuroso 2021 [27]	Japan	Cervical posterior decompression surgery	256	NR	184	PNI	50	49.6	Standard value	Age, body mass index	Complications, SSI	1 month	7
Acarbaş 2021 [31]	Turkey	All spine surgeries	454	215	70.7	PNI CONUT GNRI	45.4 5 92	NR	ROC	Age, coronary artery disease, chronic renal disease, history of malignancy, preoperative hemoglobin, creatinine, albumin, and C-reactive protein levels,	Complications	NR	7
Watanabe 2020 [29]	Japan	All spine surgeries	270	109	NR	GNRI	92	11.5	Standard value	NR	Complications	1 month	7
Oe 2020 [24]	Japan	Spinal deformity surgery	414	52	68	PNI	50	41.4	Standard value	Gender, body mass index, age, operative time, operative blood loss, days to ambulation, medical history of malignant disorder, cardiovascular disorder, and Carlson Comorbidity index.	Complications, SSi	1 month	7

ASA, American Society of Anesthesiologists; NR, not reported; NOS, Newcastle Ottawa Scale; PNI, prognostic nutritional index; CONUT, controlling nutritional status; GNRI, Geriatric nutritional risk index; SSI, surgical site infection; ROC, receiver operating characteristics; ECOG, Eastern Cooperative Oncology Group

### PNI

**Fig. 2:**
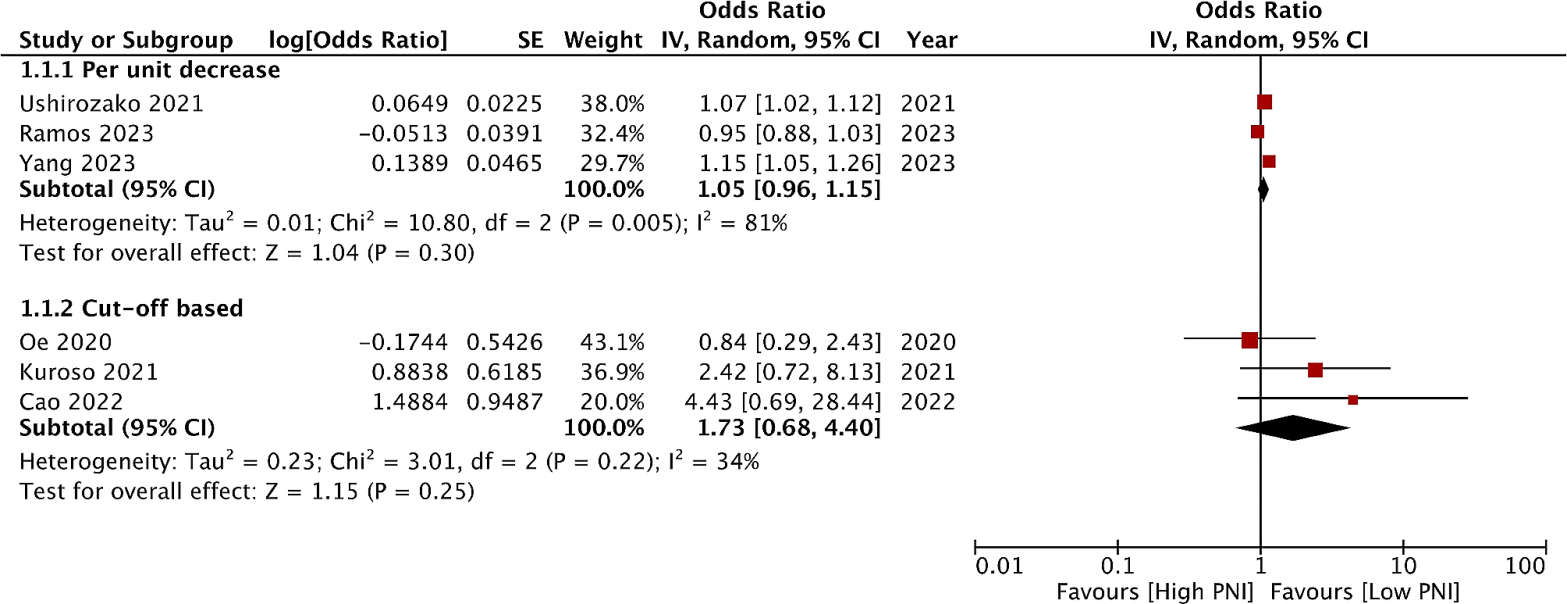
Meta-analysis of the association between PNI and SSI

**Fig. 3:**
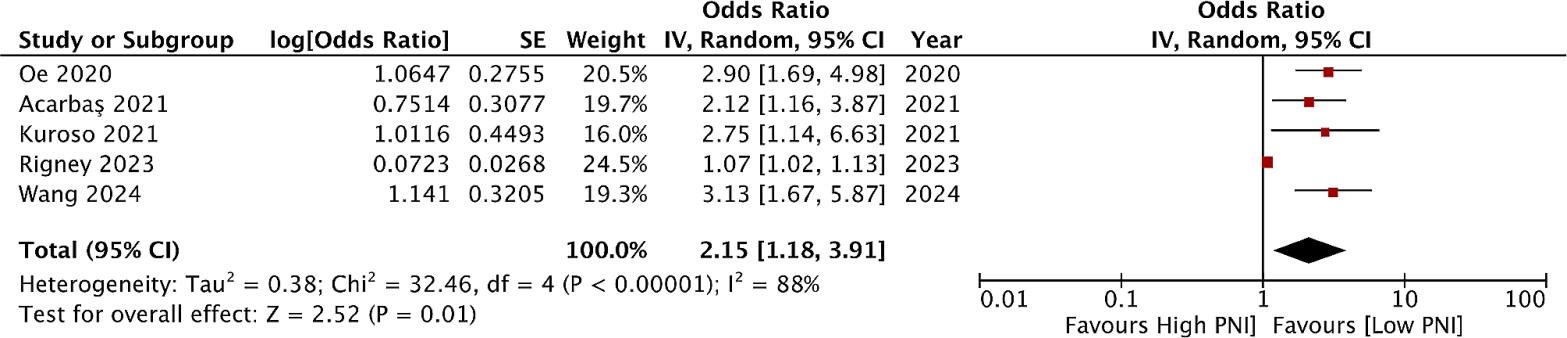
Meta-analysis of the association between PNI and adverse events

Six studies reported an association between PNI and SSI. Three reported based on per unit decrease while three used cut-off values (Fig. 2). Meta-analysis showed that a per unit decrease in PNI was not associated with an increased risk of SSI after spinal surgery (OR: 1.05 95% CI: 0.96, 1.15 I^2^ = 81%). Similarly, based on a specific cut-off low PNI was not associated with a higher risk of SSI (OR: 1.73 95% CI: 0.68, 4.40 I^2^ = 34%). Five studies reported data on all adverse events. Low PNI was significantly associated with a higher risk of adverse events (OR: 2.15 95% CI: 1.18, 3.91 I^2^ = 88%) (Fig. 3). Results remained significant during sensitivity analysis. Only one study reported a relationship between PNI and mortality. Ramos et al [32] found that per unit increase in PNI was associated with significantly higher mortality (OR: 0.86, 95% CI 0.80–0.93).

### CONUT

**Fig. 4:**
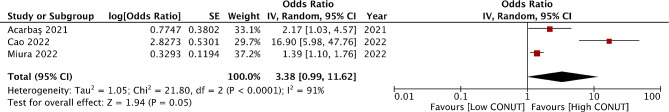
Meta-analysis of the association between CONUT and adverse events

Just three studies reported data on all adverse events. Meta-analysis showed that high CONUT was not associated with an increased risk of adverse events after spinal surgeries (OR: 3.38, 95% CI 0.99–11.62 I^2^ = 91%) (Fig. 4). One study reported data on mortality and two on SSI. Ramos et al [32] demonstrated that high CONUT was associated with a higher risk of mortality (OR: 1.70, 95% CI 1.30–2.21) but not any higher risk of SSI (OR: 1.19, 95% CI 0.92–1.55). Cao et al [26] also showed that higher CONUT did not increase the risk of SSI.

### GNRI

**Fig. 5:**
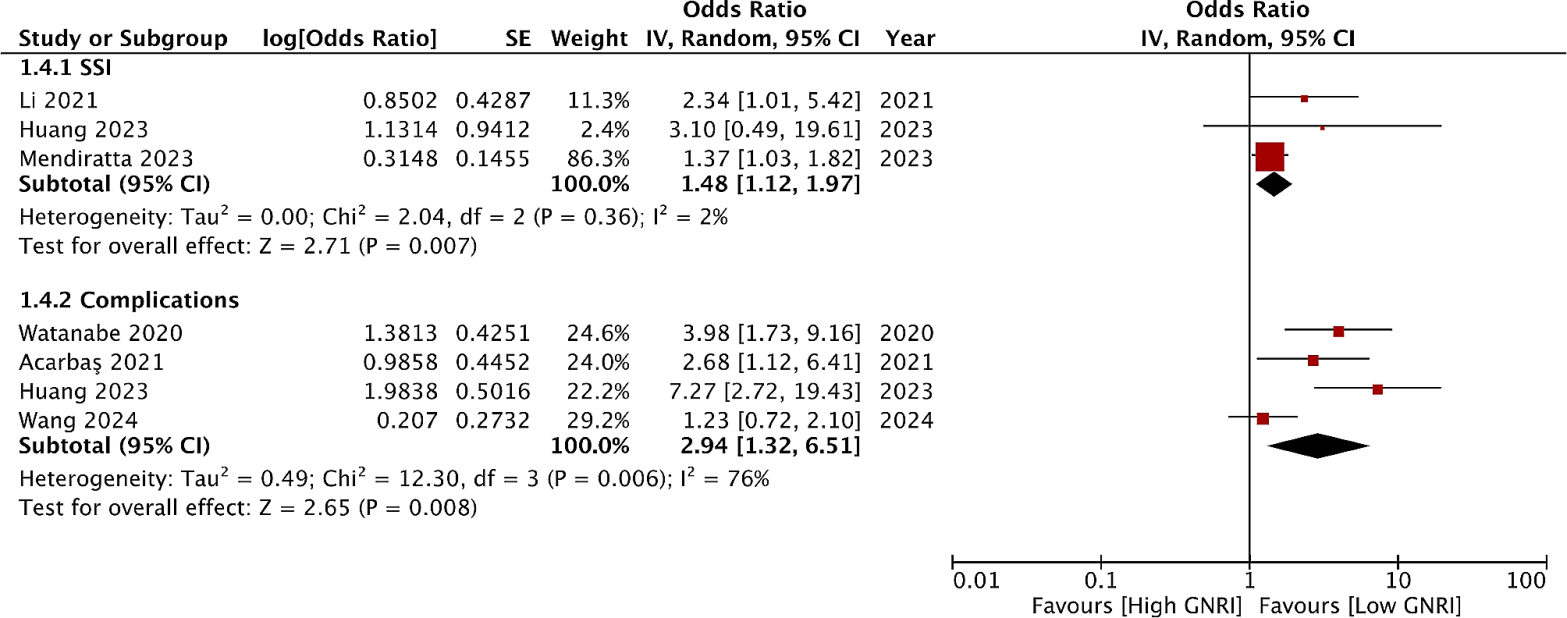
Meta-analysis of the association between GNRI and SSI and adverse events

Three studies reported data on SSI and four on overall complications. Pooled analysis showed that low GNRI was associated with increased risk of both SSI (OR: 1.48, 95% CI 1.12–1.97 I^2^ = 2%) and all adverse events (OR: 2.94, 95% CI 1.32–6.51 I^2^ = 76%) (Fig. 5). On the exclusion of Watanabe et al [29] the association between GNRI and adverse events turned non-significant (OR: 2.70, 95% CI 0.97–7.54 I^2^ = 80%). Mendiratta et al [30] also examined the risk of SSI and mortality with low GNRI. They found that low GNRI was associated with a significantly higher risk of SSI and mortality. Summary of all results of this meta-analysis is presented in Table 2.

**Table 2:** Summary of results

Index	Outcome	Number of studies	Odds ratio (95% Confidence intervals)	Conclusion
PNI (Per unit)	SSI	3	1.05 (0.96, 1.15)	Not associated with an increased risk of SSI
PNI (Cut-off based)	SSI	3	1.73 (0.68, 4.40)	Not associated with an increased risk of SSI
PNI	Adverse events		2.15 (1.18, 3.91)	Low PNI significantly associated with a higher risk of adverse events
PNI	Mortality	1	0.86 (0.80–0.93)	Increase in PNI was associated with significantly higher mortality
CONUT	Adverse events	3	3.38 0.99–11.62	Not associated with an increased risk of adverse events
CONUT	Mortality	1	1.70 (1.30–2.21)	High CONUT was associated with significantly higher risk of mortality
CONUT	SSI	2	Not pooled	Not associated with an increased risk of SSI
GNRI	SSI	3	1.48 (1.12–1.97)	High GNRI was associated with significantly higher risk of SSI
GNRI	Adverse events	4	2.94 (1.32–6.51)	High GNRI was associated with significantly higher risk of adverse events

PNI, prognostic nutritional index; CONUT, controlling nutritional status; GNRI, Geriatric nutritional risk index; SSI, surgical site infection

## Discussion

Malnutrition is highly prevalent but frequently unrecognized in a hospitalized patient. Research shows that around 45% of hospitalized patients are malnourished leading to longer lengths of stay and higher mortality [33]. Several different malnutrition markers like serum albumin, muscle mass, body mass index, Mini-Nutritional Assessment Short-Form, Subjective Global Assessment, PNI, CONUT, GNRI, etc., have been reported in the literature. Still, no single index has been found superior to others [12]. The PNI, CONUT, and the GNRI are commonly used as they are derived from routinely measured patient values thereby providing a rapid assessment of the nutritional levels of the individual. These can be easily calculated bedside thereby classifying the patient as malnourished or well-nourished allowing for appropriate interventions to improve patient outcomes. While their utility is well-defined in cancer patients [7, 8, 10], it is unclear if they can predict adverse events in patients undergoing spinal surgeries. The present study is the first meta-analysis examining the relationship between these three commonly used nutritional indices and adverse outcomes after spinal surgeries.

Collating data from a limited number of studies, we found that PNI and GNRI were significant predictors of all adverse events. Low PNI and GNRI were associated with 2x and 3x increased risk of adverse events respectively. High CONUT was also found to increase the risk of complications albeit the results did not achieve statistical significance with the lower end of 95% CI being just below 1. The low number of studies examining the association between CONUT and adverse events can be one reason for the non-significant results. Importantly, it was noted that all studies reported a positive association between PNI, CONUT, and GNRI and the risk of adverse events indicating that malnutrition is an important predictor of complications after spinal surgeries. Of the various complications after spinal surgeries, SSI is an important contributor to mortality. It is the third most adverse event with a pooled prevalence of around 3% [34]. In a separate analysis, we noted that PNI did not predict SSI after spinal surgeries, however, low GNRI was a significant predictor of SSI leading to a 1.5x increase risk of infections. Just two studies reported data on CONUT and both noted no association with SSI. Lastly, data on mortality was scarcely reported which prohibited strong conclusions.

Similar to our review, a positive association between PNI, CONUT, GNRI, and adverse outcomes has been noted for other surgical procedures as well. Zhou et al [35] have shown that PNI is a predictor of overall adverse events and infectious complications after bowel resection for Crohn’s disease. Recent research has shown that all three indices are independent predictors of mortality after hip fracture [36–38]. Yagi et al [39] in a sample of 211 patients have demonstrated that CONUT is an independent predictor of postoperative adverse events after hip fracture. Hanada et al [40] have found that PNI is predictive of increased risk of aseptic wound complications after knee arthroplasty.

Despite all three indices being associated with adverse outcomes, the superiority of one index over others has not been established. Gong et al [41] in a cohort of 167 orthopedic patients and 103 neurosurgical patients have shown that both PNI and GNRI are predictors of adverse events and longer hospital stays, but the GNRI may have a better predictive ability. In a cohort of 113 patients undergoing pancreaticoduodenectomy, Cong et al [42] have shown that all three indices were predictors of postoperative complications but PNI had the highest diagnostic efficacy. In the case of heart failure patients, both PNI and GNRI are superior to CONUT in predicting mortality [43]. Wang et al [44] have found that GNRI is the most accurate in predicting malnutrition as per the European Society of Clinical Nutrition and Metabolism (ESPEN 2015) diagnostic guidelines in esophageal cancer patients. Given the discordant results, all three nutritional indices are being used for preoperative nutritional screening of surgical patients and no single index has been universally adopted. In the current review, we were unable to analyze the sensitivity and specificity of the three indices in predicting complications due to a lack of data. Further comparative studies are needed to answer this clinical dilemma.

The PNI, CONUT, and GNRI are all nutritional screening tools but use different variables. A common variable between the three is albumin which is a widely used nutritional marker [12]. A meta-analysis of 13 studies has shown that preoperative hypoalbuminemia is a significant predictor of adverse events after spinal degenerative and deformity surgeries [45]. However, comorbid conditions and several other confounders can affect albumin levels which makes it an unreliable marker when used singularly. Hence, it has been combined with other variables to obtain more robust indices to screen for malnutrition. Lymphocytes which represent cell-mediated immunity form a part of PNI. Both humoral and cell-mediated immunity have an critical role in the systemic response to surgical injury and tissue healing. Preoperative lymphocyte levels have been associated with increased mortality and complications after surgical procedures [46]. Cholesterol and body mass index which are components of CONUT and GNRI are markers of the immune and nutritional status of the patient. They are also a part of metabolic syndrome which is associated with increased risk of surgical complications [47].

The are several limitations of this review that need to be considered while interpreting the results. Despite a detailed literature search, the quantity of studies obtained for each nutritional index was not high. There were differences in reporting of outcomes which reduced the number of studies in each meta-analysis. Data for mortality was inadequately reported which precluded a meta-analysis. Also, there were many methodological variations among studies which contributed to the high heterogeneity in the meta-analysis. The type of surgical procedures, cut-off of the nutritional index, type of complications, and follow-up had major variations that could have skewed the results. Due to a low number of studies, a subgroup analysis could not be conducted for such variations. Adverse events after any surgical procedure are dependent on numerous confounders. While adjusted data was reported by the majority of studies, the confounders analyzed differed and many unknown factors could have influenced the outcomes. Lastly, the majority of data was from a small group of countries which prevents generalization of results.

Our results indicate that a large proportion of patients undergoing spinal surgeries are malnourished and at an increased risk of adverse postoperative outcomes. While prehabilitation consisting of physical exercise, nutritional supplementation, and cognitive behavioral therapies are known to improve patient recovery and reduce complications [48], it was unclear in what subset of patients should it be prioritized. More often than not worsening symptoms and advanced disease (especially spinal oncological cases) require urgent surgery which may preclude prehabilitation. Therefore, the use of simple malnutrition tools like PNI, CONUT, and GNRI can aid in identifying high-risk individuals who could benefit from preoperative nutritional rehabilitation. Such tools should be an integral part of preoperative patient workup so that morbidity after spinal surgery is reduced especially in the malnourished population.

## Conclusions

The PNI and GNRI can predict adverse outcomes after spinal surgeries. Limited data shows that high CONUT is also associated with a non-significant increased risk of adverse outcomes. High GNRI is also associated with an increased risk of SSI. Data on mortality is too scarce for strong conclusions. Further research is required to improve current evidence.

### Electronic supplementary material

Below is the link to the electronic supplementary material.

Supplementary Material 1

## Data Availability

The authors confirm that the data supporting the findings of this study are available within the article and in its supplementary materials.
