# Supplementary material for: Do nutritional assessment tools (PNI, CONUT, GNRI) predict adverse events after spinal surgeries? A systematic review and meta-analysis
Source: J Orthop Surg Res. 2024 May 12;19:289. doi: 10.1186/s13018-024-04771-3 (PMC11089772; doi:10.1186/s13018-024-04771-3)
Supplement: Supplementary file 1 — Supplementary Material 1 [file 13018_2024_4771_MOESM1_ESM.docx]

Supplementary material 1: Search strategy and detailed search term

Database: PubMed, CENTRAL, Scopus, and Embase

Query 1. (((((((spine surgery) OR (spinal surgery)) OR (lumbar fusion)) OR (spinal tuberculosis)) OR (spinal deformity)) OR (cervical decompression)) OR (lumbar decompression)) AND (geriatric nutritional risk index)

Query 2. (((((((spine surgery) OR (spinal surgery)) OR (lumbar fusion)) OR (spinal tuberculosis)) OR (spinal deformity)) OR (cervical decompression)) OR (lumbar decompression)) AND (controlling nutritional status)

Query 3. (((((((spine surgery) OR (spinal surgery)) OR (lumbar fusion)) OR (spinal tuberculosis)) OR (spinal deformity)) OR (cervical decompression)) OR (lumbar decompression)) AND (prognostic nutritional index)
